# Preventive Cardiology in the Digital and COVID-19 Era: A Brave New World within the Veterans Health Administration

**DOI:** 10.3390/healthcare9121623

**Published:** 2021-11-24

**Authors:** Sohil Khanna, Arash Harzand

**Affiliations:** 1 School of Osteopathic Medicine, Rowan University, Stratford, NJ 08084, USA; 2 Division of Cardiology, Emory University School of Medicine, Atlanta, GA 30322, USA; aharzan@emory.edu; 3 Atlanta Veterans Affairs Medical Center, Decatur, GA 30033, USA

**Keywords:** COVID-19, telemedicine, telerehabilitation, cardiac rehabilitation, cardiovascular diseases

## Abstract

The past year challenged patients, health care providers, and health systems alike to adapt and recalibrate to meet healthcare needs within pandemic constraints. The coronavirus 2019 (COVID-19) pandemic has radically interfered with the accessibility and delivery of cardiovascular care in the United States. With an emphasis on social distancing and stay-at-home orders in effect, many Americans delayed seeking routine medical care and treatment for acute cardiac symptoms due to fear of contracting the coronavirus. The COVID-19 pandemic compelled a rapid shift toward virtual care solutions across cardiovascular domains. The U.S Department of Veterans Affairs (VA) expanded virtual modalities, notably in specialty care and rehabilitation, which offered secure solutions to maintain treatment continuity. Within the VA and other health systems, virtual cardiac rehabilitation (CR) was embraced as an efficacious alternative to on-site cardiac rehabilitation that enabled patients to receive cardiac care remotely. Leveraging the infrastructure and lessons learned from the pandemic-induced expansion of virtual care carries enormous potential to refine virtual CR and revitalize future treatment paradigms for cardiovascular disease patients.

## 1. Introduction

**Figure 1:**
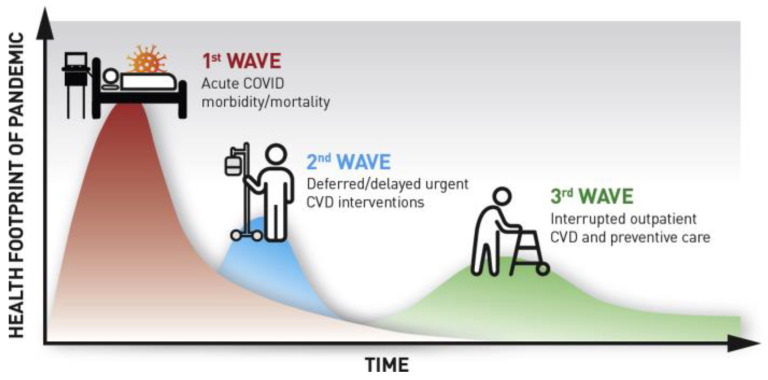
Implications of Delay and Disruption of Care for Patients with and at Risk for Cardiovascular Disease During the COVID-19 Pandemic (Am J Prev Cardiol. 2020 Mar; 1: 100009).

The U.S Department of Veterans Affairs (VA) is the nation’s largest healthcare system serving more than 9 million Veterans across 170 hospitals and over 100 outpatient facilities. As the coronavirus 2019 (COVID-19) pandemic spread through the U.S. in early 2020, the VA began deferring routine care and elective procedures alongside an unprecedented national shift towards virtual care [1]. This transition was especially pervasive within the cardiovascular care continuum, as postponed interventions and decreased acute care hospitalizations early in the pandemic gave way to extended delays in preventive and routine outpatient cardiac care (Figure 1) [2]. Between March and April, 2020, an estimated 42% fewer patients were admitted to VA inpatient facilities, with significant reductions in hospitalizations for myocardial infarction (40%), heart failure (49%), and stroke (52%), as patients elected to largely avoid hospitals in order to minimize their risk of COVID-19 exposure [3].

As an early adopter of telehealth programs beginning in the 1990s, the VA had existing bandwidth to expand virtual care offerings, yet most of the available virtual care capacity was centered within the primary care and behavioral health service lines at the start of 2020. Virtual care programs for medical specialties, including cardiology, were far less prevalent until COVID-19 abruptly forced widespread expansion beginning in mid-March 2020 [1]. The VA rapidly scaled the availability of virtual care options for Veterans with cardiac and specialty needs in an unprecedented manner over the ensuing months [1]. This paper aims to review this collective experience from the dramatic expansion of virtual cardiac services during the COVID-19 pandemic and to make recommendations for clinicians and hospital leaders to sustain these practices for future viability.

## 2. Proliferation of Virtual Care

In early 2020, VA providers and front-line staff were catapulted into novel territory when COVID-19 precipitated a sudden suspension of most in-person care, which led to a forceful expansion of virtual care offerings. On 15 March 2020, national VA leadership advised all VA hospitals and outpatient facilities to defer non-emergent care and convert to virtual care where possible [1]. As a nationwide integrated health system, the VA was tasked with maintaining care for a patient population across an expansive geographic area (including all 50 states, 4 U.S. territories, and the Philippines) with contrasting local infection levels, availability of telehealth-trained healthcare staff, and varying levels of technology access among patients and providers, including broadband and mobile device availability.

Despite these challenges, the VA was able to increase the total number of weekly video-to-home encounters from 10,695 to 137,335 across all specialties nationwide from March to April 2020. Within specialty care services, including cardiology, weekly video encounters increased from 1238 to 21,215 [1]. Overall, specialty care experienced a 14.2-fold increase in video-based encounters in the first three months of the pandemic, compared to a 6.4-fold increase for mental health care and a 15.6-fold increase in primary care encounters [4]. Rehabilitation care experienced the largest increase in phone-based encounters with a 4.8-fold increase compared to mental health care (4.5-fold), specialty care (2.9-fold), and diagnostic care (1.3-fold) [4]. Additionally, the VA marshalled mobile apps and virtual tools, including text-messaging protocols through Annie App, a digital chatbot. Annie App brought asynchronous chronic disease management to high-risk Veterans by facilitating Veteran self-care and providing remote patient monitoring (RPM) programs for hypertension and heart failure.

Existing virtual care users within the VA both prior to and during the pandemic were more likely to be non-White, Hispanic, single, disabled status, and urban dwelling compared to Veterans who had never previously engaged with virtual care. New users of video encounters during the pandemic were more likely to be female, reside in urban settings, struggle with homelessness, and suffer from a disability status. Age also offers a compelling lens into virtual care utilization, as Veterans over the age of 45 were substantially less likely to use video care in the pandemic period compared to Veterans aged 18–44 [4]. Overall, the uptick in virtual care utilization within the Veteran population can be stratified and examined across various demographics and populations.

## 3. Expansion of Virtual Cardiac Rehabilitation

COVID-19 immediately halted 71% of on-site cardiac rehabilitation (CR) programs across the US, further deepening the chasm between available cardiac services and the needs of the cardiac population [5]. Even before the pandemic, fewer than 40 facilities within the VA have historically provided access to an on-site CR program. To address this, the VA began implementing a virtual CR program in 2010 to expand CR availability to Veterans who did not reside near an on-site CR program [6]. As of early 2020, 25 VA medical centers had delivered home-based cardiac rehabilitation (HBCR) to more than 4,000 Veterans with strong evidence of effectiveness and safety in a wide range of patients including those with high complexity and clinical risk [7].

Beyond the VA, many hospital systems across the U.S. turned towards virtual CR for care continuity for their high-risk cardiac populations. The Cleveland Clinic Health System developed a teleCRehab program to integrate virtual care into their existing on-site CR program by using a tiered, algorithmic approach for classifying cardiac risk and identifying which patients are best suited for a virtually-delivered CR program [8]. Striving for technological equity, the teleCRehab program allowed weekly virtual sessions to occur via multiple modalities including smartphones, basic cellular telephones, or landlines. While this limited the ability to provide continuous audiovisual feedback during home-based exercise for each patient, the device flexibility enabled broader patient engagement beyond the digital divide with no adverse events reported [8]. Now, more than a year later, the teleCRehab program has demonstrated early promise for scaling virtual CR, having grown from the main Cleveland Clinic campus to eight regional hospitals [8].

**Figure 2:**
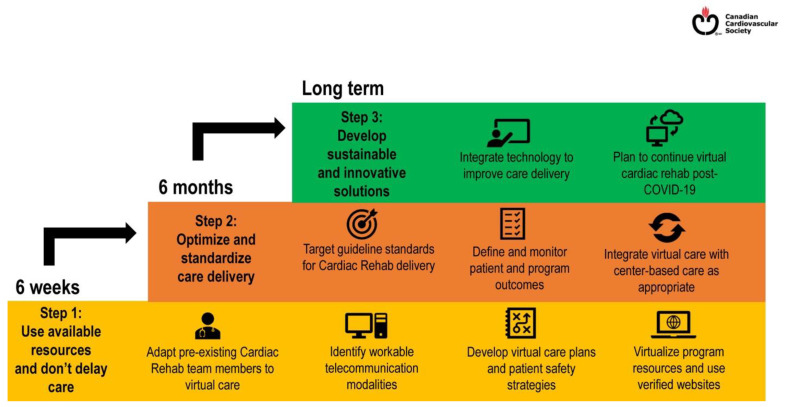
Steps of Virtual Cardiac Delivery (Cardiac Rehabilitation During the COVID-19 Era: Guidance on Implementing Virtual Care. Canadian Journal of Cardiology. 2020/08/01/ 2020; 36(8): 1317–132).

Internationally, the global impact of the pandemic on traditional CR programs has been significant. Approximately 4,400 programs across 70 countries either shut down or temporarily ceased delivering services during COVID-19, reflecting a near sudden cessation of about 70% of the global CR capacity [9]. Despite this, many of countries outside the US were able to quickly pivot towards providing virtual services [9]. Similar to VA, the United Kingdom (UK), Canada, and Australia have all been early leaders in HBCR based in large part on national health system sponsorship and supportive reimbursement policies in many of these countries [10]. All three countries have national healthcare coverage policies that endorse and cover both center-based cardiac rehabilitation (CBCR) and HBCR for eligible patients [10]. In Canada, for example, virtual CR was significantly underutilized prior to the pandemic and the Canadian Cardiovascular Society (CCS) provided recommendations for rapid deployment of virtual CR in a manner similar to that utilized in the United States (Figure 2) [11]. The CCS guidelines were notable in their inclusion of a broad spectrum of Canadian health system types, from fully integrated systems with prior virtual CR experience to hospitals with no prior experience utilizing virtual CR.

In the UK, rates of virtual CR increased more than threefold during the pandemic – from 22% to 72% [12]. Further, across Asia, a recent meta-analysis on technology-assisted CR in China concluded that virtual CR can serve as a potential alternative to traditional cardiac rehabilitation by removing barriers to care [13]. Successful implementation across countries validates virtual CR as a feasible and impactful solution to meet the needs of cardiac patients worldwide.

While virtual and home-based forms of CR were established before COVID-19, a lack of available reimbursement models had limited widespread adoption beyond integrated and full risk-bearing systems, such as the VA and Kaiser Permanente [6]. COVID-19 provided a catalyst for regulators and health systems to support expansion of virtual forms of CR to offer care for many of the highest-risk cardiac patients [6]. In the U.S., the Centers for Medicare & Medicaid Services (CMS) approved reimbursement for HBCR beginning on 14 October 2020, in an effort to increase the supply of CR during the pandemic [14]. The CMS Hospitals Without Walls initiative expanded reimbursement for over 80 additional telehealth services, broadened remote monitoring services for COVID-19 and chronic conditions, and extended the Medicare Home Health Benefit based on clinician assessments of COVID-19 exposure [15]. The number of Medicare beneficiaries using telemedicine services grew from 13,000 patients during the week before the COVID-19 pandemic to nearly 1.7 million patients per week in the last week of April 2020 [16]. Reimbursement hurdles directly influenced practice patterns, as many HBCR programs were leveraging remote physiological monitoring Current Procedural Terminology (CPT) codes for virtual CR [2]. The shift to virtual care in the context of COVID-19 further highlighted the need for well-integrated, interdisciplinary teams, as the virtual setting allowed multiple members of the care team to interact with a patient during a single visit. Many commercial HBCR vendors waived fees during peak pandemic months, which dovetailed with providers’ ability to recommend virtual CR as a treatment option when indicated [2]. 

A large urban multi-site health system explored practice patterns and patient outcomes to assess the effects of remote cardiac care on guideline-directed medical therapies (GDMT), emergency department visits, hospitalizations, and mortality [17]. Their results indicated clinicians ordered significantly fewer tests, specifically electrocardiograms, stress echocardiograms, and laboratory tests, per visit in the COVID-19 era compared with pre-COVID-19 visits. There was a further decrease in test ordering when comparing remote visits to in-person visits within the COVID-19 era [17]. Providers prescribed fewer *β*-blockers, mineralocorticoid receptor antagonists, nitrates, hydralazine, and loop diuretics during video visits compared to in-person visits for patients with heart failure and an ejection fraction less than 35% [17]. When comparing telephone to in-person visits, providers were significantly less likely to prescribe any GDMT [17]. In the COVID-19 era, patients utilizing video encounters compared with those seen in-person did not have a significant difference in rates of 90-day Emergency Department (ED) visits, hospitalizations, length of stay, or mortality rates. Telephone visits in the COVID-19 era were associated with reductions in diagnostic test ordering, GDMT prescription, increasing trends in 90-day mortality, and increases in heart failure ED visits [17].

Although published data on virtual CR referrals and uptake across the U.S. remains limited, anecdotal reports suggest that many medical centers began offering access to CR services through various mechanisms [5]. Several U.S.-based clinical trials assessing rates of virtual CR adoption and associated outcomes during COVID-19 are currently underway. Deploying virtual CR, while simultaneously advocating for reimbursement and policy changes, will be necessary to provide a sustained path forward for health systems to maintain and expand CR services going forward.

## 4. Conclusions

The direct impact of the COVID-19 pandemic has been monumental; however, there are substantial ongoing concerns about indirect effects, particularly on long-term prevention and management for patients with heart disease. While focused on Veterans in remote and rural areas, VA’s prior investment in technology, human resources, and policy related to telehealth provided a firm foundation for the rapid and widespread acceleration of virtual cardiac services, such as virtual CR, that COVID-19 demanded. Beyond VA, expanded reimbursement for telehealth services and significant limitations on in-person care were fundamental drivers extending virtual services during the pandemic. It remains unclear whether this significant growth in virtual care will endure, especially as many of the exclusions that enabled increased telehealth services under the federal public health emergency (PHE) may be sunset by the end of 2021.

One critical output of the pandemic was the growth of standardized virtual CR programs for identifying high-risk cardiovascular patients and deploying remote secondary prevention protocols. Cardiac rehabilitation has been shown to reduce hospital readmission by 25% and death by 42%, yet only a quarter of eligible patients were participating in CR prior to the pandemic [5]. The availability of virtual CR ensured that cardiovascular patients received ongoing preventive services despite an increasingly overwhelmed healthcare system. Despite the interim progress, a universal system for delivering virtual CR has still not been embraced. Maintaining remote CR programs beyond the pandemic would enable health systems to leverage the benefits of CR for larger populations while further refining delivery methods, processes, and outcome measures to provide even more value.

Virtual CR has tremendous potential to transform cardiovascular care and revolutionize cardiovascular recovery. The advantages of a virtual CR program include capturing a greater portion of the patient population eligible for CR, decreasing barriers to patient participation, encouraging better patient self-care, improving care delivery to patients in remote areas, and enhancing the patient journey and overall satisfaction by delivering care in the patient’s home or community. The associated cost-savings from a combination of reduced infrastructure expenditures, reduction in spending on preventable complications, and better human resource and staff allocation only work to further enrich the cost-benefit calculus of adopting virtual CR. 

Meanwhile, a comprehensive understanding of potential disadvantages of virtual approaches to CR is needed to help facilities and their leadership teams design programs that align with specific patient needs, financial considerations, and resource allocation. While virtual CR undoubtedly supported many patients with cardiac care needs during the height of the pandemic, the distribution of benefits for virtual care were not enjoyed proportionally. Within VA, older, homeless, and rural Veterans have been less likely to engage in virtual visits in general, which raises considerable concern for access barriers plaguing these populations [4]. The incongruency in responsiveness and adoption among both patient populations and provider groups may have hindered the overall efficacy and pace of virtual care deployment early in the pandemic. From a health system perspective, the potential disadvantages of virtual CR include the cost associated with acquiring, scaling, and revamping telehealth capabilities, technological constraints and interoperability with existing information technology infrastructure, and uncertainty related to reimbursement and regulatory policy changes as many of the pandemic-era waivers begin to expire. 

Although these considerations in implementing or scaling virtual CR programs warrant reflection, the benefits afforded are multi-factorial and the opportunity cost of not embracing virtual CR is simply too steep. The comprehensive positive impact and opportunities to strategically design virtual programs with hybrid combinations of remote and virtual care, tailored to the needs of a given site, contribute to the case for maintaining and scaling virtual CR in a post-COVID-19 era. Now that many systems have expanded virtual services including CR, future efforts should be directed towards ensuring the population accessing CR continues to grow both in diversity and scope. In this regard, the COVID-19 pandemic may have served as a watershed moment to recognize the potential behind virtual cardiac care programs, particularly preventive services, for the future.

## Data Availability

No new data were created or analyzed in this study. Data sharing is not applicable to this article.
